# Supplementary material for: Head-Space SPME for the Analysis of Organophosphorus Insecticides by Novel Silica IL-Based Fibers in Real Samples
Source: Molecules. 2022 Jul 22;27(15):4688. doi: 10.3390/molecules27154688 (PMC9330742; doi:10.3390/molecules27154688)
Supplement: Supplementary file 1 [file molecules-27-04688-s001.zip › molecules-1805870-supplementary.pdf]

Supplementary Materials

Head-space SPME for the analysis of organophosphorous insecticide by silica IL-based fibers in real samples

Karolina Delińska [a,*], Kateryna Yavir [a], Adam Kloskowski [a]

[a] Gdansk University of Technology, Faculty of Chemistry, Department of Physical Chemistry, Narutowicza Str.11/12, Gdansk 80-233 Poland

** Corresponding author e-mail address: karolina.delinska@pg.edu.pl (K. Delińska)*

Table S1. Physicochemical properties of the studied insecticides.

compound	structure	pKa value	molecular weight [g/mol]	CAS number	$logK_{ow}$	purity [%]
heptenophos	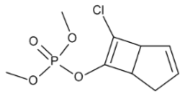	2.72	250.61	23560-59-0	2.32	≥ 98.0
dimethoate	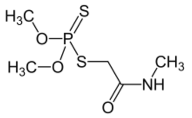	0.78	229.3	60-51-5	0.78	≥ 98.0
diazinon	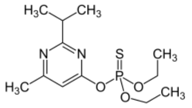	3.11	304.35	333-41-5	3.30	≥ 98.0
paraoxon ethyl	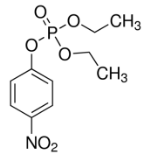	1.98	275.19	311-45-5	1.98	≥ 95.0
fenitrothion	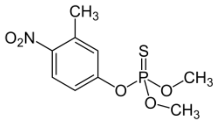	n.a.	277.23	122-14-5	3.30	≥ 95.0
chlorfenvinphos	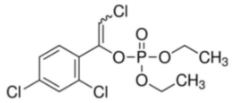	4.70	359.6	470-90-6	3.81	~ 90%
phosalone	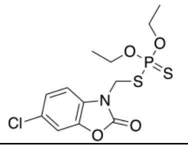	4.38	367.81	2310-17-0	4.38	≥ 98.0

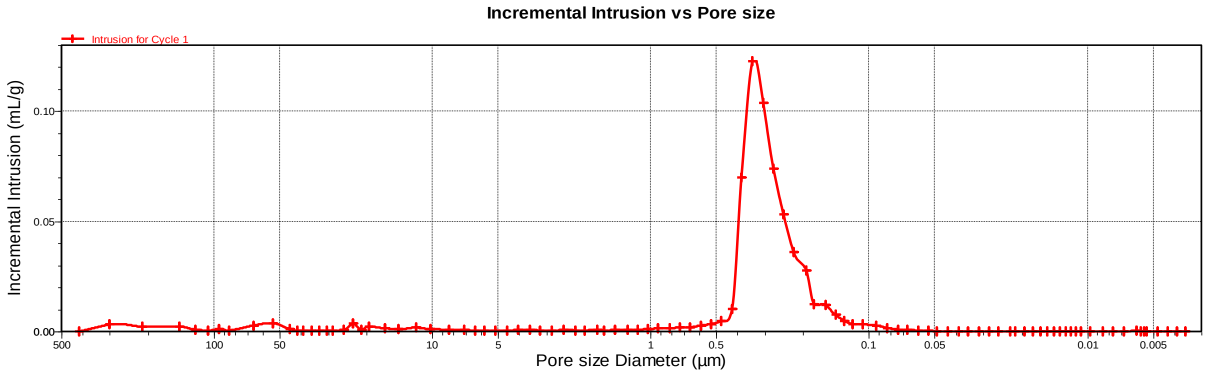

Figure S1. Mercury Intrusion Porosimetry: Plot of incremental intrusion vs. pore size. The measurement was performed with the use of AutoPore IV 9500 V1.09 Micrometrics Instrument Corporation.

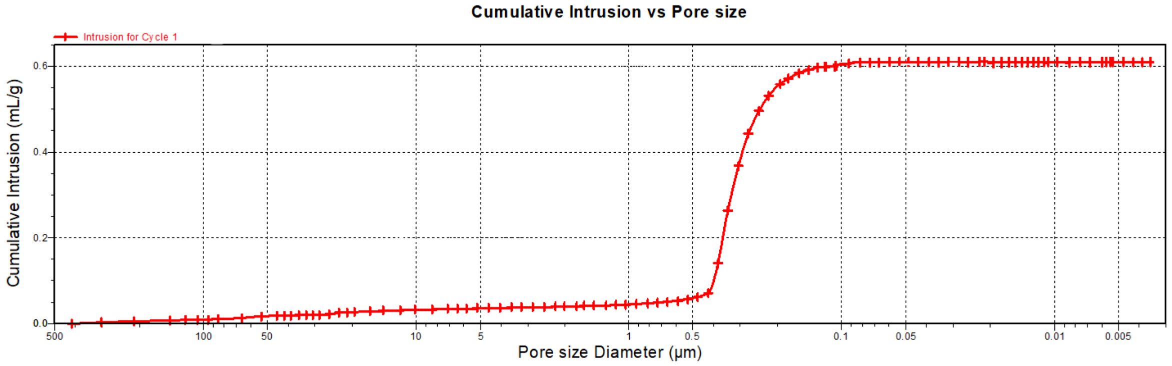

Figure S2. Mercury Intrusion Porosimetry: Cumulative intrusion vs. pore size. The measurement was performed with the use of AutoPore IV 9500 V1.09 Micrometrics Instrument Corporation.

Table S2. Physicochemical properties of the studied ionic liquids.

Ionic liquid (IL)	IL structure	Acronym of IL	MW	Tm	Td	d	η	CAS
1-Butyl-1-methylpyrrolidinium bis(trifluoromethylsulfonyl) imide	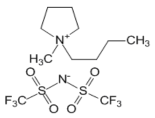	IL-1	422.4	<RT	360	1.40	95	223437-11-4
1-Benzyl-3-methylimidazolium bis(trifluoromethylsulfonyl)imide	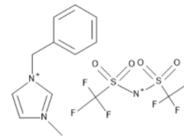	IL-2	208.69	<RT	396	1.49	135	433337-24-7
1-(2-Methoxyethyl)-3-methylimidazolium bis(trifluoromethylsulfonyl) imide	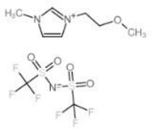	IL-3	421.34	<RT	358	n.a.	46.9	178631-01-1
Butyltriethyl ammonium bis(trifluoromethylsulfonyl) imide	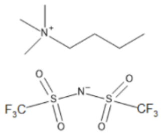	IL-4	369.4	<RT	>220	1.39	99.5	258273-75-5

[a] MW – molecular weight;
[b] T_m – melting point[ºC];
[c] T_d – decomposition temperature[ºC];
[d] d – density $[g{*}cm^3]$;
[e] η – viscosity [cP];
CAS – CAS number

Table S3. CCD plan and responses (sum of the peak areas), which were obtained to extract the insecticides with the investigated IL-2 based fiber.

No	System (CCD)	Extraction time [min]	Equilibration time [min]	Temperature [ºC]	Salt concentration [%]	pH	Chromatographic peak area
**1**	25	70	50	60	15	3	5468
**2**	20	70	80	60	15	7	9366
**3**	14	90	65	50	20	5	10935
**4**	13	90	65	50	10	9	9941
**5**	31 (C)	70	50	60	15	7	8129
**6**	9	90	35	50	10	5	5965
**7**	32 (C)	70	50	60	15	7	7921
**8**	8	50	65	70	20	5	6416
**9**	30 (C)	70	50	60	15	7	8745
**10**	7	50	65	70	10	9	4447
**11**	17	30	50	60	15	7	4474
**12**	36 (C)	70	50	60	15	7	8153
**13**	1	50	35	50	10	9	3872
**14**	33 (C)	70	50	60	15	7	8986
**15**	26	70	50	60	15	11	7459
**16**	29 (C)	70	50	60	15	7	8201
**17**	22	70	50	80	15	7	9941
**18**	2	50	35	50	20	5	7570
**19**	19	70	20	60	15	7	9861
**20**	21	70	50	40	15	7	4971
**21**	12	90	35	70	20	5	9444
**22**	18	110	50	60	15	7	11432
**23**	28 (C)	70	50	60	15	7	8651
**24**	34 (C)	70	50	60	15	7	8742
**25**	35 (C)	70	50	60	15	7	7987
**26**	15	90	65	70	10	5	10935
**27**	6	50	65	50	20	9	7099
**28**	4	50	35	70	20	9	8947
**29**	3	50	35	70	10	5	5064
**30**	16	90	65	70	20	9	11929
**31**	10	90	35	50	20	9	6959
**32**	23	70	50	60	5	7	4474
**33**	11	90	35	70	10	9	8947
**34**	27 (C)	70	50	60	15	7	9341
**35**	5	50	65	50	10	5	3067
**36**	24	70	50	60	25	7	12128

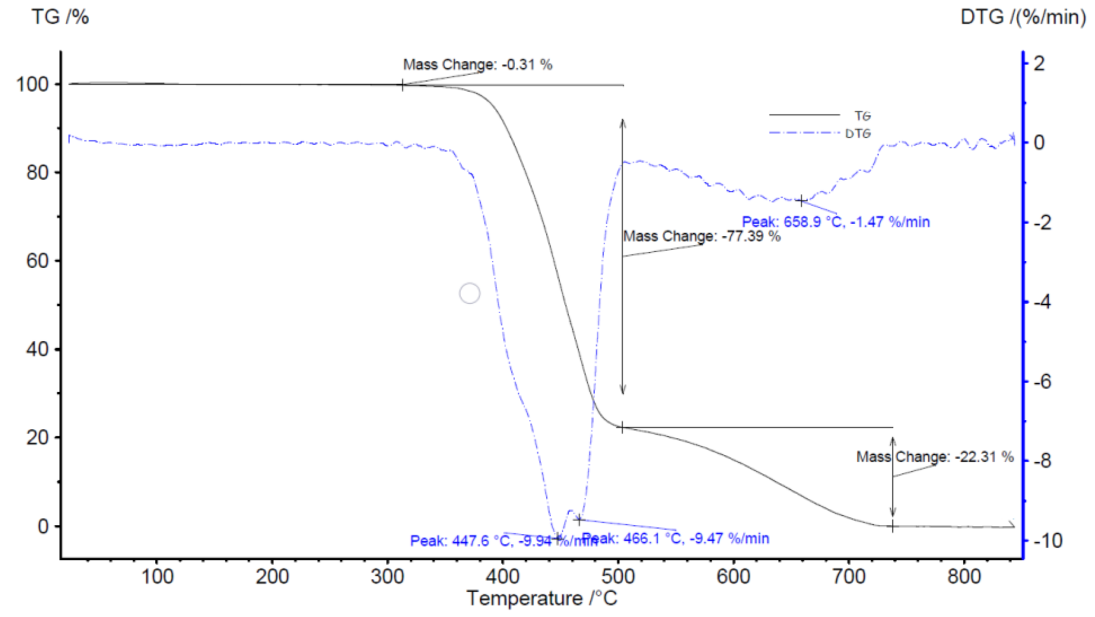

Figure S3. Thermogravimetric analysis of pure ionic liquid, namely 1-Benzyl-3-methylimidazolium bis(trifluoromethylsulfonyl) imide.

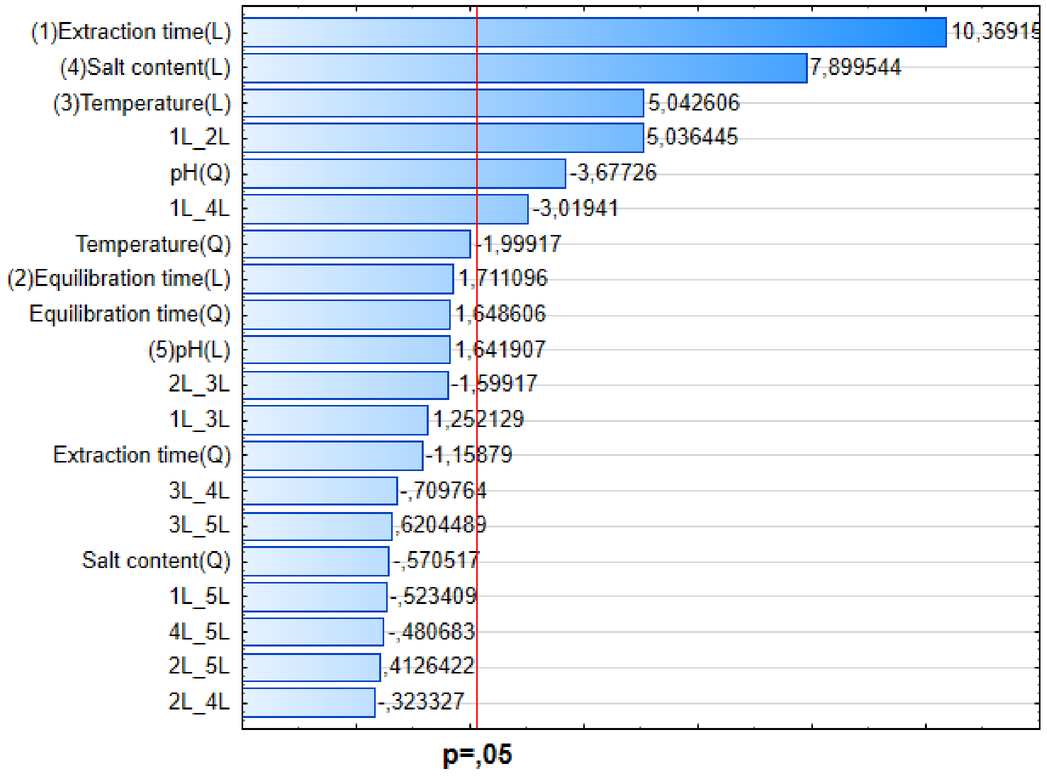

Figure S4. Statistical significance of the effects of the extraction parameters on extraction performance of the IL-based fiber: standardized effect Pareto chart.
